# A Basal Tapejarine (Pterosauria; Pterodactyloidea; Tapejaridae) from the Crato Formation, Early Cretaceous of Brazil

**DOI:** 10.1371/journal.pone.0162692

**Published:** 2016-09-21

**Authors:** Rodrigo Vargas Pêgas, Maria Eduarda de Castro Leal, Alexander Wilhelm Armin Kellner

**Affiliations:** 1 Laboratory of Systematics and Taphonomy of Fossil Vertebrates, Departamento de Geologia e Paleontologia, Universidade Federal do Rio de Janeiro, Museu Nacional, Rio de Janeiro, Brazil; 2 Departamento de Geologia, Universidade Federal do Ceará, Fortaleza, Ceará, Brazil; Institute of Botany, CHINA

## Abstract

A three-dimensional and almost complete pterosaur mandible from the Crato Formation (Early Cretaceous of Northeastern Brazil), Araripe Basin, is described as a new species of a tapejarine tapejarid. Tapejarines are a particular group of toothless pterosaurs, characterized by well-developed cranial crests, downturned rostra, and have been proposed to represent frugivorous flying reptiles. Though comparatively well represented and distributed, the evolutionary history of the group is still poorly known, and the internal relationships of its members are not well understood. The new species here reported, named *Aymberedactylus cearensis* gen. et sp. nov., adds new data concerning the evolution of the group, concerning their morphology and geographical origin. It differs from known tapejarids due to its unusually elongate retroarticular process and a shallow fossa on the splenial exhibiting distinctive rugose texture. Furthermore, it exhibits a suite of basal and derived conditions within the Tapejaridae, demonstrating how their morphological traits probably evolved and that these forms were even more diverse than already acknowledged. The discovery of *Aymberedactylus cearensis* sheds new light on the evolutionary history of the Tapejarinae.

## Introduction

The Tapejaridae are a clade of toothless pterosaurs easily recognized by their well-developed cranial sagittal crests and particularly large nasoantorbital fenestrae [1–5]. The clade has been split into the subfamilies Thalassodrominae and Tapejarinae: whereas thalassodromine tapejarids display straight dorsal jaw margins and premaxillary crests that extend very high posteriorly, tapejarines typically display short, downturned jaws and premaxillary crests anteriorly high that constrict posteriorly, and well-developed dentary crests [5]. The Tapejarinae include at least ten species ranging from the Barremian to the Turonian-Campanian [6, 7], and displaying a widespread geographic distribution, having been found in Brazil, China, Morocco and Spain [8]. These pterosaurs typically display an occlusal gap and a step-like dorsal margin of the dentary symphyses, traits that have been interpreted as linked to a frugivore diet [9]. The presence of fruits in the diet of tapejarines has been subsequently accepted by several workers [7, 8, 10–13].

The Thalassodrominae, in contrast, are much less diverse, with a very narrow geographic distribution that could suggest an endemic pattern. So far, all described and confirmed species come from the Romualdo Formation (Aptian-Albian, Santana Group, Araripe Basin): *Tupuxuara longicristatus*, *Tupuxuara leonardii* and *Thalassodromeus sethi* [6]. A fourth thalassodromine species from the same formation, “*Tupuxuara deliradamus*”, has been proposed [14], and later invalidated [6]. Another described species, *Lacusovagus magnificens* from the Crato Formation (Aptian, Santana Group, Araripe Basin), was assigned to the Chaoyangopteridae [15], but could be a thalassodromine [6, 16].

So far, pterosaur taxa from the clades Anhangueria (*sensu* Rodrigues & Kellner, 2013 [17] and Azhdarchoidea (*sensu* Kellner, 2003 [2]) are known from the Crato Formation. The former are represented by *Brasileodactylus araripensis* [18] and *Ludodactylus sibbicki* [19], whereas the latter are represented by the tapejarine tapejarids *Tupandactylus imperator* [20] and *Tupandactylus navigans* [21], plus the indeterminate azhdarchoid *Lacusovagus magnificens*. All of those are known from isolated cranial material. Over three hundred indeterminate postcranial specimens from the Crato Formation have been assigned to the same clades [12].

Here we report a new species of pterosaur from the Crato Formation, based on a single three-dimensional lower jaw. In virtue of its toothlessness, slightly downturned dentary symphysis and vestiges of a dentary crest, it can be identified as a tapejarine. It displays a unique combination of thalassodromine-like and tapejarine features, which together suggest its placement as a basal tapejarine. This inference is corroborated by a phylogenetic analysis presented here. The new species exhibits a novel morphology among its relatives and provides new insights into tapejarid diversity and tapejarine evolution.

## Geological Setting

The Santana Group of Araripe Basin, located in Northeastern Brazil, records the transgression-regression cycle taking place in the Afro-Brazilian rift system during the Aptian-Albian worldwide marine transgression [22–25]. It contains two of the most important Mesozoic fossil *Konservat Lagerstätte* on Gondwana, the Crato and Romualdo Formations [22–26].

The Crato Formation comprises mostly micritic laminated limestone rocks, interpreted as deposits from the shallow waters of a coastal lagoon with both marine and fluvial influences [23]. Although its fossils are generally compressed to some level, these beds are famous for yielding exceptionally preserved remains [23, 26], including a pterosaur patagium [27], soft tissue crests [20, 21, 28], rhamphothecae [20, 21, 28], and possible pycnofibers [28]. Furthermore, possible muscle fibers of a turtle [29] and several feathers [30–33] are preserved, some of them having even revealed melanosomes [34].

## Materials and Methods

### Phylogenetic Analysis

In order to assess the phylogenetic position of *Aymberedactylus cearensis*, we coded it in a data matrix modified from [7], itself based on previous works [2, 3, 8], and ran a phylogenetic analysis using the software TNT [35], default traditional search. We modified a character (52) by splitting the original state in two. The original derived state refers to a mandibular symphysis that accounts for over 30% of mandibular length. We restricted state (1) to such proportion between 30–60% and created state (2) as 60% and over. See Supporting Information (List A in S1 File) for more detail. We also added a new character (51) concerning mandible width. This latter feature was assessed by dividing the distance between the articular cotyles of each mandibular ramus (including their own widths; see Figure A in S1 File) by the mandibular length, measured from the articular cotyle to the rostral tip. We chose the distance between the two articular cotyles instead of the distance between the tips of the two retroarticular processes because such a feature can be inferred using the skull for taxa with unknown complete mandibles, based on the distance between the condyles of the quadrates and the distance between the quadrates and the rostral premaxillary tip. We also corrected the scoring of character 61 as given by [7], which is related to serrated teeth and was miscoded for some toothless taxa.

### Nomenclatural acts

Under the amended International Code of Zoological Nomenclature (ICZN), the online version of this article conforms to the requirements for the availability of the new names contained here. This published work and its nomenclatural acts have been registered in the online registration system of the ICZN, the ZooBank. The ZooBank LSIDs (Life Science Identifiers) can be resolved and the associated information viewed through any standard web browser by appending the LSID to the prefix “http://zoobank.org/”. The LSID for this publication is: urn:lsid:zoobank.org:pub:AA4278E7-DE1A-4F52-94FA-4A0E0DADEAAB.

The specimen herein described (MN 7596-V) is housed in the Museu Nacional/Universidade Federal do Rio de Janeiro. No field work was carried out. No permits were required for this study, which complied with all regulations. The specimen was donated to the Museu Nacional/UFRJ. There are no specific coordinates. The information available is: found near the quarries of the city of Nova Olinda, Ceará, Brazil.

### Institutional Abbreviations

AMNH: American Museum of Natural History, New York, USA; CP: Centro Paleontológico (Universidade do Contestado), Mafra, Rio Grande do Sul, Brazil; CPCA: Centro de Pesquisas Paleontológicas da Chapada do Araripe (Departamento Nacional de Produção Mineral), Crato, Brazil; GMN: Geological Museum of Nanjing, China; HGM: Henan Geological Museum, Zhengzhou, China; IMCF: Iwaki Coal and Fossil Museum, Japan; IMNH: Iwaki Museum of Natural History, Japan; LPM: Liaoning Paleontological Museum, China; M: Zhejiang Museum of Natural History, Hangzhou, Zhejiang, China; MCCM: Las Hoyas collection of the Museo de las Ciencias de Castilla—La Mancha, Cuenca, Spain; MN: Museu Nacional (Universidade Federal do Rio de Janeiro), Rio de Janeiro, Brazil; TMM: Texas Memorial Museum (University of Texas), Austin, USA.

## Results

### Systematic Paleontology

**Systematic hierarchy.**

Pterosauria Kaup, 1834

Pterodactyloidea Plieninger, 1901

Azhdarchoidea Nessov, 1984

Tapejaridae Kellner, 1989

Tapejarinae Kellner & Campos, 2007

*Aymberedactylus cearensis* gen. et sp. nov.

**ZooBank Life Science Identifier (LSID) for genus:** urn:lsid:zoobank.org:act:CFF72AD6-6472-4290-9C58-AE85AA3CC12D

**ZooBank LSID for species:** urn:lsid:zoobank.org:act:0D69853A-AA21-478C-B50B-9F6B273CF92D

**Etymology:** The generic name is a combination of *aymbere*, meaning “small lizard” in the Tupi language (one of the main Brazilian indigenous cultures), and *dactylus*, from the Greek word *daktylus* for “finger”, a commonly used suffix for pterodactyloids. The specific epithet refers to Ceará, Brazilian state of provenance of the fossil.

**Fig 1:**
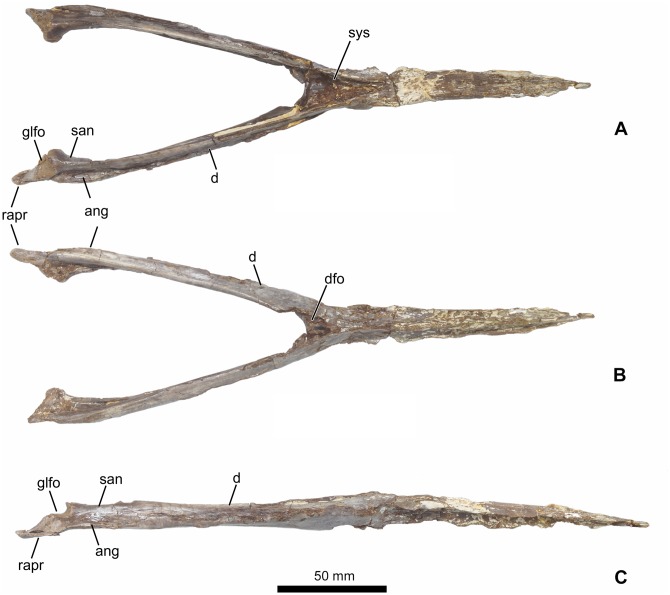
Holotype of *Aymberedactylus cearensis* gen. et sp. nov. (MN 7596-V). (A) Dorsal view. (B) Ventral view. (C) Right lateral view. Abbreviations: ang = angular, d = dentary, dfo = dentary fossa, glfo = glenoid fossa, rapr = retroarticular process, san = surangular, sys = symphyseal shelf.

**Holotype:** MN 7596-V, almost complete mandible (Fig 1).

**Locality and horizon:** Specimen from near the quarries of Nova Olinda city, Ceará, northeastern Brazil. Crato Formation, Araripe Basin, Lower Cretaceous (Aptian-Albian).

**Diagnosis:** The new species displays the following autapomorphies: elongated retroarticular process (approximately 7% of total mandibular length); shallow fossa on the splenial exhibiting distinctive rugose texture.

It can be further differentiated from other azhdarchoid species by the following combination of features: deep symphyseal shelf, dorsally concave mandibular symphysis throughout entire extent, wide lower jaw (0.34 ratio of mandibular width/length), shallow and short dentary fossa, and an accentuate angle of divergence between mandibular rami and symphysis of ~165°(angle between the two rami of ~30°).

### Description and Comparisons

The specimen consists of a three-dimensionally preserved mandible, with a total length of 270 mm. It is virtually complete, except for the left retroarticular process and part of the dentary crest (Fig 1). The mandible is completely edentulous.

**Fig 2:**
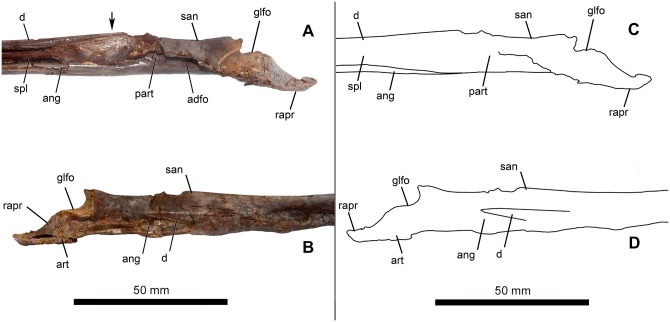
Right mandibular ramus of *Aymberedactylus cearensis* gen. et sp. nov. (MN 7596-V). (A) Medial view. Arrow indicates rugose surface. (B) Lateral view. (C) Schematic drawing of the ramus in medial view. (D) Schematic drawing of the ramus in lateral view. Abbreviations: adfo = adductor fossa, ang = angular, art = articular, d = dentary, glfo = glenoid fossa, part = prearticular, rapr = retroarticular process, san = surangular, spl = splenial.

The largest element is the dentary, as is true for pterosaurs. The two dentaries are anteriorly fused, forming a mandibular symphysis that accounts for approximately 50% of total mandibular length. This compares well with most tapejarine species and contrasts with the other known edentulous pterosaurs clades, namely the Thalassodrominae, Azhdarchidae, Chaoyangopteridae, Pteranodontidae and Nyctosauridae (Table 1). A faint suture can be seen between the dentary and the surangular (Fig 2A), while a well-defined suture separates the splenial from the angular (Fig 2B). The articular surface for the quadrates exhibit a rugose, rough texture.

**Table 1:** Comparative selected mandible ratios of various pterosaurs.

Clade	Lower jaw/Taxa	Symphysis length/ total mandibular length	Jaw width/ jaw length	Retroarticular process length/ total mandibular length	Reference
Tapejarinae	*Aymberedactylus cearensis* gen. et sp. nov.	50%	0.34	0.07	This study
Tapejarinae	*Tapejara wellnhoferi*	38%	0.30	0.05	[9]
Tapejarinae	*Tupandactylus imperator*	51%	?	0.02	[28]
Tapejarinae	*Caiuajara dobruskii*	46%	0.49	?	[7]
Tapejarinae	*Caupedactylus ybaka*	~65%	~0.18	?	[6]
Thalassodrominae	*Tupuxuara leonardii*	60%	0.20	0.04	[4]
Azhdarchoidea	*“Tupuxuara deliradamus”*	?	?	0.01	[14]
Azhdarchoidea	*Bakonydraco galaczi*	50%	0.22	0.029	[11]
Chaoyangopteridae	*Shenzhoupterus chaoyangensis*	~60%	?	~0.04	[43]
Chaoyangopteridae	*Chaoyangopterus zhangi*	~60%	?	?	[52]
Chaoyangopteridae	*Jidapterus edentus*	~61%	?	?	[47]
Azhdarchidae	*Quetzalcoatlus* sp.	~60%	0.11	0.023	[42]
Azhdarchidae	*Zhejiangopterus linhaiensis*	~60%	?	~0.03	[41]
Pteranodontidae	*Pteranodon longiceps*	68%	0.14	0.03	[38]
Nyctosauridae	*Nyctosaurus* cf. *gracilis*	62%	0.20	?	[40]

*Aymberedactylus* can also be set apart from two other toothless pterosaurs from Araripe Basin. It differs from the azhdarchoid *Lacusovagus magnificens* due to the latter’s unique S-shaped upper jaw margins [15], which most likely suggests a complementary shape for its mandible (but see [36] for exceptions). *Aymberedactylus* also differs from *Banguela oberlii*, from Romualdo Formation, by the lack of a blade-like mandibular symphysis and an upturned rostral mandibular tip that are seen in the latter [37].

**Fig 3:**
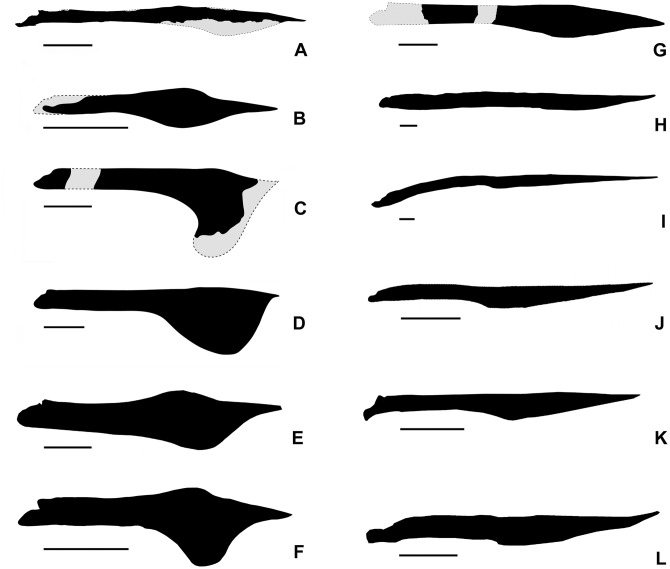
Comparison of azhdarchoid mandibles (right lateral view). (A) *Aymberedactylus cearensis* gen. et sp. nov. (MN 7596-V). (B) *Sinopterus dongi* (GMN-03-11-001, holotype of “*Huaxiapterus jii*”, based on [8, 46]). (C) *Europejara olcadesorum* (MCCM-LH 9413, based on [8]). (D) *Tupandactylus imperator* (CPCA 3590, based on [8, 28]. (E) *Caiuajara dobruskii* (CP.V 1005a, based on [7]). (F) *Tapejara wellnhoferi* (IMNH 1053, based on [13]). (G) *Caupedactylus ybaka* (MN 4726-V; based on [6]). (H) *Tupuxuara leonardii* (IMCF 1052, based on [3]). (I) *Quetzalcoatlus* sp. (TMM 42161–2, based on [42]). (J) *Zhejiangopterus linhaiensis* (M 1330, based on [41]). (K) *Shenzoupterus chaoyangensis* (HGM 41HIII- 305A, based on [43]). (L) *Chaoyangopterus zhangi* (LPM-R00076, based on [52]). All scales are 50 mm in length.

The dorsal margin of the mandibular symphysis of *Aymberedactylus* is curved downwards, apparently to a lesser extent than what is seen in tapejarines such as *Caupedactylus ybaka*, *Tapejara wellnhoferi* or *Caiuajara dobruskii* (Fig 3). It is likely that, in life, the new taxon exhibited a somewhat more pronounced curvature of the mandibular symphysis, which would had been distorted by taphonomical dorsoventral compression. This curvature also differentiates *Aymberedactylus* from pteranodontids [38, 39], nyctosaurids [40], azhdarchids [41, 42] and thalassodromines [3], while tapejarines typically display ventrally deflected dentary symphyses [8]. Among chaoyangopterids, *Chaoyangopterus zhangi* displays a dentary slightly curved upwards [10], while the same bone is apparently curved slightly downwards in *Shenzhoupterus chaoyangensis* (Fig 3K) [43].

**Fig 4:**
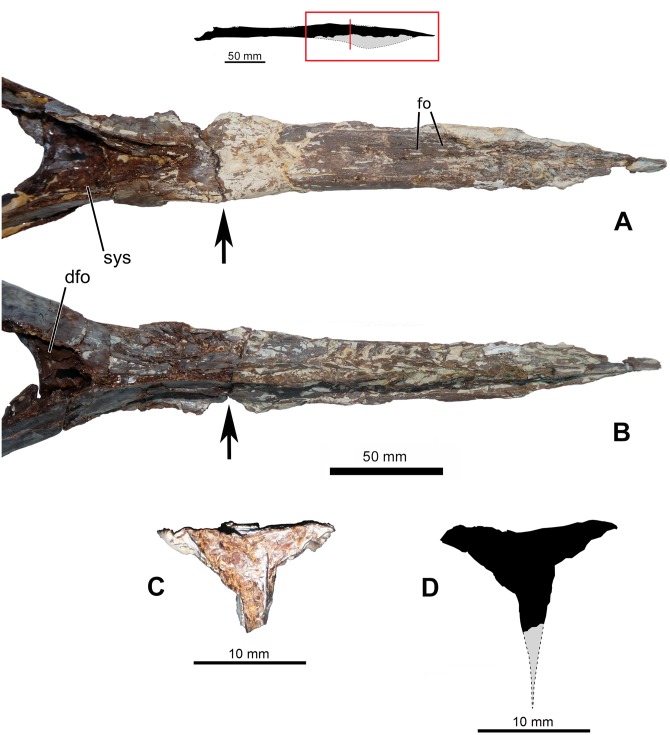
Mandibular symphysis of *Aymberedactylus cearensis* gen. et sp. nov. (MN 7596-V). (A) Reconstruction of the mandible of *Aymberedactylus cearensis*, with a red box indicating the zoomed areas and a red line indicating the depicted cross-section. (B) Mandibular symphysis in dorsal view. (C) Mandibular symphysis in ventral view. Arrows indicate a fracture that reveals the cross-section. (D) Cross-section. (E) Reconstructed cross section. Abbreviations: dfo = dentary fossa, fo = foramen, sys = symphyseal shelf.

Small neurovascular foramina can be seen on the posterodorsal region of the dentary symphysis (Fig 4B), anteriorly to the symphyseal shelf, indicating that the region might have been covered by a horny sheath as in *Tupandactylus imperator* [25]. Similar foramina have been reported in the jaws of several azhdarchoid species [1, 7, 11, 44, 45].

Though the ventral surface of the dentary symphysis is damaged, the anterior and posteriormost ventral margins of a crest can still be seen (Fig 4C). The outline of these remaining original ventral edges and the width of the broken bases of the crest suggest that the dentary crest was shallow (Fig 4D–4E), deeper than the ridge-like condition of *Tupuxuara leonardii* [3] but probably shallower than the derived tapejarines *Tapejara*, *Tupandactylus*, *Europejara* and *Caiuajara* [7–9, 28]. Such crest was possibly similar to the crest seen in *Sinopterus dongii* [46] or *Caupedactylus ybaka* [6]. This too suggests closer proximity to tapejarines than to other azhdarchoids.

The concave dorsal surface of the mandibular symphysis in dorsal view extends from the very rostral tip until the posterior end of the symphysis, where it continues to a deep symphyseal shelf (Fig 4B). The symphyseal shelf in *Aymberedactylus* is deep and displays a flat floor (Fig 4B), similarly to the condition found in *Caupedactylus ybaka*, *Thalassodromeus sethi* and *Tupuxuara leonardii*, although in the two latter taxa the shelf is much longer (see Table 1). The symphyseal shelf is much shallower for *Tapejara wellnhoferi*. *Caiuajara dobruskii*, in turn, lacks a noticeable symphyseal shelf.

**Fig 5:**
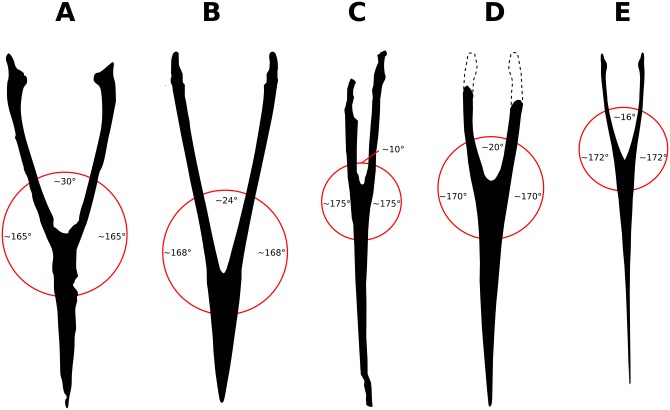
Selected pterosaur mandibles in dorsal view. (A) *Aymberedactylus cearensis* gen. et sp. nov. (MN 7596-V). (B) *Tapejara wellnhoferi* (AMNH 24440, based on [9]). (C) *Quetzalcoatlus* sp. (TMM 42161–2, based on [42]). (D) *Jidapterus edentus* (holotype, based on [47]). (E) *Pteranodon longiceps* (after [38]).

The posterior half of the dorsal surface of the mandibular symphysis displays approximately parallel lateral margins, from where the mandibular rami suddenly expand posteriorly (Fig 1). The long axis of each mandibular ramus forms an angle of ~165°with the long axis of the mandibular symphysis, with an angle of ~30°between the two rami. These features render the mandible a pronounced Y-shape. Such a shape is unique, contrasting with the usual condition see in tapejarids and pterosaurs in general of gradually divergent lateral mandibular margins, which renders the mandible rather V-shaped. The V-shaped mandibles are seen, for instance, in azhdarchids as *Quetzalcoatlus* sp. [42]; tapejarids as *Tapejara wellnhoferi* [9], *Thalassodromeus sethi* [44] and *Caupedactylus* [6]; chaoyangopterids as *Jidapterus edentus* [47]; or pteranodontoids as *Pteranodon* [38] or *Anhanguera* [48] (see Fig 5 for some examples).

The dentary fossa in *Aymberedactylus* is very short and shallow, invading only little of the interior space of the mandibular symphysis anteriorly when compared to, for example, *Caupedactylus* or *Thalassodromeus*. Its original length cannot be fully assessed due to the damaged margins of the ventral edge. It is important to remark that dentary fossae have so far only been reported for azhdarchoid pterosaurs [6].

The adductor fossa is elongate (15 mm), very thin (2–3 mm) and shallow (Fig 2B). Anterior to the adductor fossa, on the splenial, an elliptical shallow depression can be seen on both mandibular rami, exhibiting a finely rugose texture (Fig 2B). No Meckelian fossa can be seen.

Posteriorly, the articular surface for the quadrate is rather smooth, and is not segmented into two cotyles. This condition is similar to *Bakonydraco galaczi* [11] and *Tapejara wellnhoferi*, and different from *Quetzalcoatlus* sp. [42], *Thalassadromeus sethi* and *Tupuxuara leonardii*, in which medial and distal cotyles can be set apart.

The retroarticular process is unusually elongated when compared to other taxa. This structure accounts for 7% of total mandibular length in *Aymberedactylus*, over twice the same ratio for *Tupandactylus imperator*, *Thalassodromeus sethi* or *Quetzalcoatlus* sp. (Table 1).

Laterally, the rami are relatively shallow, with a preserved 0.058 ramus height/mandibular length ratio. In life, considering distortion, we estimate the real ratio may have been close to 0.075. The mandibular width:length ratio for the new specimen is 0.34, closer to some tapejarines than to other pterodactyloids (Table 1). This low width:length ratio is partly due to the relatively short mandibular symphysis of tapejarines compared to some other pterodactyloids.

### Phylogenetic Analysis Results

The inclusion of *Aymberedactylus* in the original phylogenetic analysis by [7] resulted in a strict consensus tree (of 6 most parsimonious trees) which recovered it as an indeterminate tapejarid, in a trichotomy with the Tapejarinae and the Thalassodrominae.

We remark that, prior to the inclusion of the new species, we corrected the scoring of the character 61 (relative to absence/presence of serrated teeth), which was scored as present for some toothless taxa. This did not influence the original topology by [7]. However, when the new species was added without such correction, the whole Tapejaride clade was collapsed in a polytomy, except for the small clade *Tapejara* + *Europejara* + *Caiuajara* + *Tupandactylus*.

Because mandibular symphyses proportions are one of the main characters discussed here when comparing *Aymberedactylus* to other edentulous pterosaurs, we modified character 51, relative to mandibular symphysis proportions (see SI). We also included a new character, concerning mandible broadness, which we have observed to be broader in tapejarines than in other pterodactyloids (Table 1) as discussed above.

**Fig 6:**
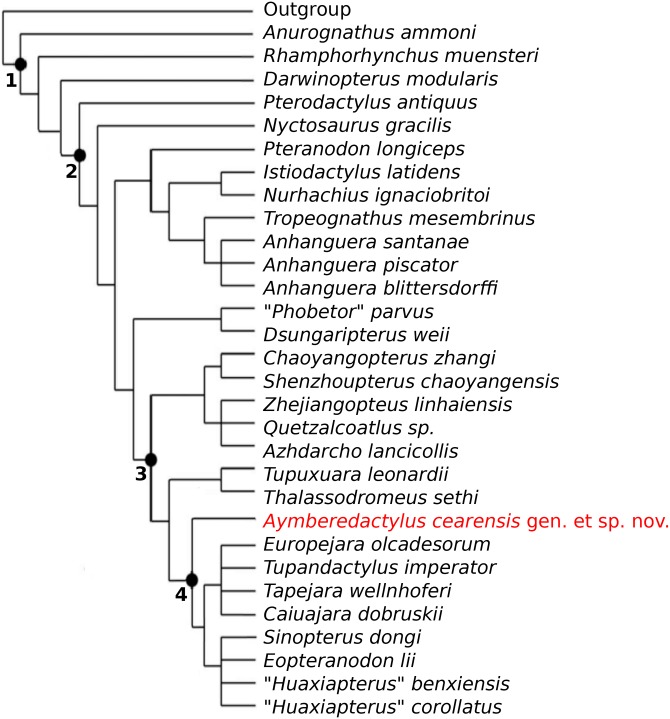
Phylogenetic analysis. Modified version (see text) of the matrix by [7], with the inclusion of *Aymberedactylus cearensis* gen. et sp. nov. Nodes: 1, Pterosauria; 2, Pterodactyloidea; 3, Azhdarchoidea; 4, Tapejarinae.

Our new phylogenetic data matrix thus generated a better resolution, with the recovery of *Aymberedactylus* as a basal tapejarine tapejarid in the strict consensus tree (of 5 most parsimonious trees; Fig 6). All 5 most parsimonious trees exhibited 231 steps, a consistency index of 0.688 and a retention index of 0.829.

*Aymberedactylus* and other tapejarines share as an ambiguous synapomorphy a mandibular symphysis corresponding to 30–59% (actually 38–51% in analyzed taxa) total mandibular length. It is interesting to observe that, while ambiguous within the Pterosauria, this synapomorphy becomes unambiguous relative to the Azhdarchoidea alone, thus indicating a secondary shortening of the mandibular symphysis within the Tapejarinae. This indicates that an elongated mandibular symphysis is general and plesiomorphic for the Azhdarchoidea.

The step-like dorsal margin of the dentary in lateral view, previously recovered as an unambiguous synapomorphy of the Tapejarinae [8] becomes a synapomorphy of a less inclusive clade containing all other tapejarines, excluding *Aymberedactylus*. Such clade includes Chinese tapejarines, which form a monophyletic group, and a clade formed by *Tapejara wellnhoferi* + *Europejara olcadesorum* + *Tupandactylus imperator* + *Caiuajara dobruskii*. By definition, the Tapejarinae include all tapejarids closer to *Tapejara wellnhoferi* than to *Thalassodromeus sethi* [5]. In this way, being the sister-group to these other tapejarines, *Aymberedactylus* should be interpreted as the most basal known taxon of the group.

Such topology interestingly suggests that a midway step-like convexity interrupting the concave surface of the mandibular symphysis is a derived feature of a particular group of tapejarine tapejarids, excluding *Aymberedactylus* gen. et sp. nov. and *Caupedactylus ybaka*. The presence of deep symphyseal shelves, in turn, seems to be plesiomorphic for the Tapejaridae, being present in thalassodromines and *Aymberedactylus*, being intermediate in the tapejarine *Caupedactylus* and much shallower in the other tapejarines.

## Discussion

Recognizing specific ontogenetic stages in pterosaurs is rather complicated, and most criteria rely largely on postcranial features (e.g. [48–50]), all of which are unknown for *Aymberedactylus cearensis*. Nonetheless, the degree of fusion of the bones of MN 7596-V indicates that it does not represent a juvenile (e.g. [48–50]). However, because some sutures can be seen, it does not represent a fully mature individual either, likely representing a subadult (e.g. [48–50]). The rough, rugose texture of the articular surface for the quadrates is similar to the condition seen in the articular surfaces of the long bones of subadult specimens attributed to *Pteranodon* [49]. This condition is indicative of incomplete ossification [49].

We estimate a relatively small wingspan for *Aymberedactylus*, of approximately 2 meters, as scaled from the mandibles of other tapejarines such as *Tapejara wellnhoferi* [9], *Europejara olcadesorum* [8] and *Caiuajara dobruskii* [7]. This compares well with the typical wingspan of adult tapejarines [3, 7, 8]. Given that MN 7596-V is deemed here a subadult based on the persistence of sutures between the dentary and the surangular, and between the splenial and the angular, we cannot know for sure the wingspan that *Aymberedactylus cearensis* could have achieved when fully grown. However, the degree of bone fusion is compatible with a late subadult, making it unlikely that it could have grown to reach the typical wingspans of around 4 meters of thalassodromines [3]. In the case of *Pteranodon*, subadults exhibit nearly to exactly the same size as their adult forms, from which they cannot be set apart based on size-related criteria [49].

Among pterosaurs, toothlessness is a condition found in the Pteranodontidae, Nyctosauridae and Azhdarchoidea. *Banguela oberlii* is a purported toothless dsungaripterid from Romualdo Formation [37], though it lacks any unambiguous synapomorphies of the Dsungaripteridae and shares several features with *Thalassodromeus sethi*, to which the only known specimen was originally referred to [51]. All azhdarchoid clades—the Azhdarchidae, Chaoyangopteridae and Tapejaridae (Tapejarinae + Thalassodrominae; sensu Kellner & Campos, 2007 [5])–are edentulous [43]. Among these, elongate mandibular symphyses accounting for at least 60% of total mandibular length are found in the Pteranodontidae [38], Nyctosauridae [40], Azhdarchidae [41, 42], Chaoyangopteridae [43, 52] and Thalassodrominae (Table 1). The same is true for the probable basal tapejarine *Caupedactylus ybaka* [6], though not for other known tapejarines. The shorter mandibular symphyses within toothless pterosaurs (50% of total jaw length and under) are found in the Tapejarinae, with the exception of *Caupedactylus ybaka* [3, 6] (see Table 1). *Bakonydraco galaczi* also exhibits a mandibular symphysis that accounts for half of the total mandibular length, though it is not yet well established whether this form represents an azhdarchid as originally proposed [11] and later accepted [13, 53] or a tapejarine, as recently reinterpreted according to phylogenetic analyses [54, 55]. The proportion of the mandibular symphysis of *Aymberedactylus* therefore indicates a relationship to tapejarine pterosaurs. Additionally, the specimen herein described exhibits a dentary fossa, structure so far only reported for azhdarchoids [6], reinforcing the azhdarchoid nature of *Aymberedactylus*.

Current knowledge of the pterosaurian mandible therefore indicates *Aymberedactylus cearensis* gen. et sp. nov. to represent a basal tapejarine. This interpretation is corroborated by our phylogenetic analysis. Its toothless mandibular symphysis corresponding to half of total mandibular length and its shallow dentary crest suggest tapejarine affinities, while the lack of a step-like dorsal margin of the mandible in lateral view indicate a basal position within the group. The deep symphyseal shelf, shared with thalassodromines, seems to be a feature later attenuated throughout more derived tapejarines.

*Aymberedactylus* is also similar to non-tapejarine azhdarchoids in respect to the dorsal surface of the mandibular symphysis in dorsal view. Known tapejarines such as *Tapejara wellnhoferi* (Fig 3F), “*Huaxiapterus*” *benxiensis* and *Caiuajara dobruskii* (Fig 3E) display a mid-way convex dorsal surface on the dentary symphysis, which is located posterior to a small concavity and anterior to the symphyseal shelf [7, 9, 56]. Such convexity gives the mandible of these tapejarines a step-like dorsal margin in lateral view, and this is particularly prominent in *Tapejara wellnhoferi* and *Caiuajara dobruskii*. Due to this convexity, a gap is formed between the jaws during occlusion (e.g. [9]). “*Huaxiapterus*” *benxiensis* seems to exhibit such morphology as well, though in this taxon the anterior concavity (the shallow groove, as described) seems not to extend until the very anterior tip [56]. *Tupandactylus imperator* and *Europejara olcadesorum* also display concavities confined to the rostral mandibular tip, though the condition present posteriorly cannot be clearly assessed [8, 28]. In the basal tapejarine *Caupedactylus ybaka*, an anterior concavity is also present, separated from the symphyseal shelf by a short flat surface, instead of a strong step-like convexity [6]. *Aymberedactylus*, on the other hand, displays a continuously concave dorsal margin of the whole mandibular symphysis in dorsal view. This pattern is similar to the thalassodromine *Tupuxuara leonardii*, whereas the unique and unusual mandible of *Thalassodromeus sethi* displays a blade-like morphology with a sharp dorsal keel. The Azhdarchidae, in turn, seem to display distinct configurations, though more investigation on the azhdarchid mandible is needed. In *Quetzalcoatlus* sp. and *Alanqa saharica*, a concave dorsal surface is present on the mandibular symphysis, though it does not extend anteriorly until the very anterior tip of the dentary symphysis, which is actually flat [42, 45]. *Volgadraco bogolubovi* also exhibits a dorsal concavity on the mandibular symphysis, though the tip is broken and cannot be assessed [57]. In *Azhdarcho lancicollis*, in turn, the dorsal surface of the dentary symphysis displays a flat surface as well, though the anterior concavity extends until the very tip [58].

Another likely trend in tapejarine evolution is the deepening of the lateral profile of their mandibular rami. The lateral depth of the mandibular rami of *Aymberedactylus* is intermediate between *Tupuxuara leonardii*, with a ratio of 0.06, the basal tapejarine *Caupedactylus ybaka*, with a ratio of 0.05 [6], and other taxa with deeper mandibular rami, such as a ratio of 0.08 for *Sinopterus* [59] and *Europejara* [8]; 0.085 for *Tupandactylus imperator* [28] and over 0.1 for *Tapejara wellnhoferi* [9] and *Caiuajara dobruskii* [7]. The same value is 0.03 for the azhdarchid *Quetzalcoatlus* sp. [42]. See Fig 3 for a comparison between the new species and several azhdarchoid taxa. The downward curvature of the dentary symphysis is another possible trend, even though the exact original curvature of *Aymberedactylus* in life is unkown.

Within the Azhdarchoidea, *Aymberedactylus cearensis* is similar to thalassodromines, chaoyangopterids and azhdarchids in lacking a step-like dorsal margin of the dentary in lateral view. This feature in tapejarines such as *Tapejara wellnhoferi* and *Caiuajara dobruskii* is the result of a small convexity on the dorsal surface of the dentary symphysis, which separates an anterior concavity from the symphyseal shelf. This reinforces the basal nature of *Aymberedactylus* relative to other tapejarines, and also demonstrates that the shortening and deflection of the dentary symphysis appeared earlier than the step-like margin of the dentary on the course of the tapejarine evolutionary history.

*Aymberedactylus cearensis* is further unique in displaying a thin, lightly built Y-shaped mandible, with elongate retroarticular processes. We emphasize that we do not interpret here such feature as seen in *Aymberedactylus* as taphonomical in nature, given that previous taphonomical experiments have shown that flattening, either by decay or compression, without rock metamorphism, does not lead to significant lateral expansion of the body outline in invertebrates [60]. As for organisms with mineralized skeletons, they would rather behave in a “brittle” way, with cracks, fractures and overlapping of mineralized tissues accommodating the skeleton under compaction. Although compaction-related deformation is a well-known phenomenon in the fossil record, there are few quantitative studies on how morphology is actually distorted [61].

The thin symphysis and the spread out rami provide low resistance to shaking and torsion [62], indicating that *Aymberedactylus cearensis* was probably not capable of delivering strong bites or handling relatively large or struggling prey. The mandibular fossa, insertion site for the mandible adductor muscles *m*. *pseudotemporalis profundus*, *m*. *pseudotemporalis superficialis* and *m*. *adductor mandibulae externus profundus* [63], is small and shallow, indicating these muscles were not especially well-developed. These features would also prevent the delivery of strong bites.

The unusually elongate retroarticular process, in turn, indicates that m. *depressor mandibulae*, muscle which inserts on this region of the mandible in archosaurs [63], was relatively better developed in *Aymberedactylus* than in other pterosaurs. Because m. *depressor mandibulae* is responsible for mandibular abduction [63], we interpret that *Aymberedactylus* likely had good control of mandibular abduction and jaw opening.

Finally, the basal nature of *Aymberedactylus* as a tapejarine contributes to the discussion concerning the geographical origin of the group. Tapejarines were firstly discovered in Brazil [1, 20], later being found in North Africa [64], China [59] and Europe [8]. Chinese tapejarines have been often interpreted as successively basal taxa relative to South American species [28, 46], with Laurasia being regarded as the most likely area of origin for the group [8, 28]. The oldest records are Barremian in age and come from Europe and China, what has been argued to corroborate such interpretation [8]. However, Chinese tapejarines have been found to form a clade [7, 8] instead of a paraphyletic group, what does not provide stronger support for a Laurasian origin than for a Gondwanan one. Furthermore, it is interesting to notice that their sister-group, the Thalassodrominae, are so far restricted to South America [3]. The existence of basal tapejarines in Brazil such as *Aymberedactylus* and possibly *Caupedactylus ybaka* [6], combined with the exclusive Brazilian nature of thalassodromines, provides a good case for the reinterpretation of tapejarines as Gondwanan pterosaurs in origin. The monophyly of the Chinese tapejarines would demonstrate a single dispersion event to China. On the other hand, being *Europejara* closely related to South American tapejarines [8], a separate dispersion event would have occurred to Europe. We highlight that future findings, preferably with more complete specimens of *Aymberedactylus cearensis*, are needed to further support our phylogenetic analysis and hence this biogeographical reinterpretation.

## Supporting Information

S1 File Supporting Information 1. **List A. Phylogenetic Analysis.** Character list and data matrix. **Table A. Measurements of *Aymberedactylus cearensis* gen. et sp. nov. Figure A. Measurement of mandibular width (new character 51).**
(DOCX)
