# Supplementary material for: A Basal Tapejarine (Pterosauria; Pterodactyloidea; Tapejaridae) from the Crato Formation, Early Cretaceous of Brazil
Source: PLoS One. 2016 Sep 21;11(9):e0162692. doi: 10.1371/journal.pone.0162692 (PMC5031394; doi:10.1371/journal.pone.0162692)

A Basal Tapejarine (Pterosauria: Pterodactyloidea: Tapejaridae) from the Crato Formation, Early Cretaceous of Brazil

**Supporting Information 1**

**List A) Phylogenetic analysis, characters and character matrix.**

In order to access the phylogenetic position of *Aymberedactylus cearensis* gen. et sp. nov., we performed a phylogenetic analysis using TNT (Goloboff et al., 2003) using the default traditional search. This analysis is based on Manzig et al. (2014), with a modified character (52) and a new one (51). A strict consensus tree of this search is shown in Figure 6.

CHARACTER LIST (per anatomical region)

SKULL1. Dorsal margin of the skull: 0 - straight or curved downward 1 - concave 2 - only rostrum curved upward2. Upper and lower jaw: 0 - laterally compressed 1 - comparatively broad3. Rostral part of the skull anterior to the external nares: 0 - reduced 1 - elongated (less than half of skull length) 2 - extremely elongated (more than half of skull length)4. Rostral end of premaxillae/maxillae downturned: 0 - absent 1 - present5. Position of the external naris: 0 - above the premaxillary tooth row 1 - displaced posterior to the premaxillary tooth row6. Process separating the external nares: 0 - broad 1 - narrow7. External naris and antorbital fenestra: 0 - separated 1 - confluent forming a nasoantorbital fenestra

8. Naris and antorbital fenestra: 0 - shorter than 40% of the skull length 1 - longer than 40% of the skull length9. Posterior margin of antorbital (or nasoantorbital) fenestra: 0 - straight 1 - concave

10. Nasoantorbital (or antorbital) fenestra extending dorsal to the orbit 0 - absent 1 - present

11. Shape of the orbit:

0 - subcircular 1 - circular 2 - circular, with open ventral margin 3 - piriform (elongated)

12. Orbit comparatively small and positioned very high in the skull: 0 - absent 1 - present13. Position of the orbit relative to the nasoantorbital fenestra (naris + antorbital fenestra): 0 - same level or higher 1 - orbit lower than the dorsal rim of the nasoantorbital fenestra14. Suborbital opening: 0 - absent 1 - present

15. Lower temporal fenestra: 0 - piriform, with ventral portion wider than dorsal 1 - piriform, with dorsal portion wider than ventral 2 - reduced (slit-like)

16. Premaxillary sagittal crest: 0 - absent 1 - present

17. Premaxillary sagittal crest, position: 0 - confined to the anterior portion of the skull 1 - starting anterior to the anterior margin of the nasoantorbital fenestra, extending beyond occipital region 2 - starting at about the anterior margin of the nasoantorbital fenestra, reaching the skull roof above the orbit but not extending over the occipital region 3 - starting close or at the anterior portion of the skull and extended over the occipital region 4 - starting at the posterior half of the nasoantorbital fenestra. 5 - starting at the middle portion of the nasoantorbital fenestra, extending above the occipital region.

18. Premaxillary sagittal crest shape: 0 - striated, low with a nearly straight dorsal margin 1 - striated, high, spike-like

2 - round dorsal margin, blade-shaped3 - smooth, expanded anteriorly and forming a low rod-like extension posteriorly4 - smooth, starting low anteriorly and very expanded posteriorly

19. Expansion on the anterior part of the premaxillary sagittal crest: 0 - absent 1 - present

20. Elongated dorsal premaxillary spike-like extension 0 - absent 1 - present

21. Expansion of the premaxillary tip: 0 - absent 1 - present, with premaxillary end high 2 - present, with premaxillary end dorsoventrally flattened.22. Posterior ventral expansion of the maxilla: 0 - absent 1 - present

23. Maxilla-nasal contact 0 - broad 1 - absent

24. Free nasal process: 0 - absent 1 - present25. Free nasal process position:

0 - placed laterally 1 - placed medially

26. Free nasal process size:

0 - long, almost reaching the ventral margin of the skull 1 - short 2 - knob-like (extremely reduced)

27. Free nasal process orientation:

0 - directed anteroventrally 1 - directed ventrally

28. Foramen on nasal process: 0 - absent 1 - present29. Lacrimal extensively fenestrated 0 - absent 1 - present

30. Lacrimal process of the jugal thickness: 0 - broad 1 - thin31. Lacrimal process of the jugal inclination:

0 - inclined anteriorly 1 - subvertical 2 - inclined posteriorly

32. Pronounced ridge on the lateral side of the jugal 0 - absent 1 - present

33. Anterior portion of the frontal rugose: 0 - absent

1 - present

34. Bony frontal crest: 0 - absent 1 - present35. Bony frontal crest, position: 0 - confined to the posterior end of the skull 1 - starting above the orbit 2 - starting on the posterior half of the nasoantorbital fenestra36. Bony frontal crest, shape:

0 - reduced and blunt 1 - short and spike-like, dorsally deflected 2 - narrow, directed posteriorly 3 - very high and broad, at least doubling the height of the skull above the orbit, directed posteriorly 4 - high, broad base and fan-shaped.

37. Bony parietal crest: 0 - absent 1 - present38. Bony parietal crest shape: 0 - blunt 1 - constituting the base of the posterior portion of the cranial crest.39. Posterior region of the skull rounded with the squamosal displaced ventrally: 0 - absent 1 - present40. Position of the quadrate relative to the ventral margin of the skull: 0 - vertical or subvertical 1 - inclined about 1204 backwards 2 - inclined about 1504 backwards41. Position of the articulation between skull and mandible: 0 - under the posterior half of the orbit or furtherbackwards 1 - under the middle part of the orbit 2 - under the anterior half of the orbit or further anterior42. Helical jaw joint: 0 - absent 1 - present43. Supraoccipital: 0 - does not extend backwards 1 - extends backwards44. Foramen pneumaticum piercing the supraoccipital: 0 - absent 1 - present45. Expanded distal ends of the paroccipital processes: 0 - absent 1 - present46. Palatal ridge: 0 - absent 1 - discrete, tapering anteriorly 2 - strong, tapering anteriorly 3 - strong, confined to the posterior portion of the palate47. Slight expansion of the palate close to the anterior opening of the nasoantorbital (or naris + antorbital) fenestra: 0 - absent 1 - present48. Maxilla excluded from the internal naris: 0 - absent 1 - present49. Opening between pterygoids and basisphenoid (interpterygoid opening): 0 - absent or very reduced 1 - present and larger than subtemporal fenestra 2 - present but smaller than subtemporal fenestra50. Basisphenoid: 0 - short 1 - elongated

51. Mandibular width (distance between both glenoid fossae divided by the length from glenoid fossa to rostral tip ratio; NEW): 0 – under 0.25
 1 – 0.25 and over

52. Mandibular symphysis (modified after Kellner, 2003): 0 - absent or very short 1 - present, between 30% and 59% of total mandible length 2 - present, at least ~60% of total mandible length

53. Step-like dorsal margin of the dentary in lateral view: 0 - absent 1 - present54. Anterior tip of the dentary downturned: 0 - absent 1 - present 55. Dentary bony sagittal crest: 0 - absent 1 - present56. Dentary bony sagittal crest, position: 0 - confined to the anterior third of the lower jaw 1 - extending close to the middle partion of the jaw57. Dentary bony sagittal crest shape: 0 - small projection

1 - blade-like 2 - elongated ridge-like 3 - deep, broad in lateral view 4 - shallow

58. Position and presence of teeth: 0 - teeth present, evenly distributed along the jaws 1 - teeth absent from the anterior portion of the jaws 2 - teeth confined to the anterior part of the jaws 3 - jaws toothless59. Largest maxillary teeth positioned posteriorly: 0 - absent 1 - present60. Variation in the size of the anterior teeth with the 5th and 6th smaller than the 4th and 7th: 0 - absent 1 - present61. Teeth with a broad and oval base: 0 - absent 1 - present

62. Teeth finely serrated:

0 - absent 1 - present

63. Peg-like teeth: 0 - absent 1 - present, 15 less on each side of the jaws 2 - present, more than 15 on each side of the jaws

64. Laterally compressed and triangular teeth:

0 - absent

1 - presentAXIAL SKELETON65. Atlas and axis: 0 - unfused 1 - fused66. Notarium: 0 - absent 1 - present67. Postexapophyses on cervical vertebrae: 0 - absent 1 - present68. Lateral pneumatic foramen on the centrum of the cervical vertebrae: 0 - absent 1 - present69. Midcervical vertebrae: 0 - short, sub-equal in length 1 - elongated 2 - extremely elongated70. Cervical ribs on midcervical vertebrae: 0 - present 1 - absent71. Neural spines of the mid-cervical vertebrae, height: 0 - tall 1 - low 2 - extremely reduced or absent72. Neural spines of the mid-cervical vertebrae, shape: 0 - blade-like 1 - spike-like 2 - ridge73. Number of caudal vertebrae: 0 - more than 15 1 - 15 or less

74. Caudal vertebrae with elongated zygapophyses forming rod-like bony processes: 0 - absent 1 - present

75. Proximal caudal vertebrae with duplex centra: 0 - absent 1 - presentPECTORAL GIRDLE76. Length of the scapula: 0 - subequal or longer than coracoid 1 - scapula shorter than coracoid (1 > sca/cor > 0.80) 2 - substantially shorter than coracoid (sca/cor < 0.80)77. Proximal surface of scapula: 0 - elongated 1 - sub-oval78. Shape of scapula: 0 - elongated 1 - stout, with constructed shaft79. Coracoidal contact surface with sternum: 0 - flattened 1 - oval80. Coracoidal contact surface with sternum: 0 - no developed articulation surface 1 - articulation straight or slightly concave 2 - articulation strongly concave81. Posterior expansion on articulation surface of the coracoid with the sternum: 0 - absent 1 - present

82. Deep coracoidal flange: 0 - absent 1 - present83. Broad tubercle on ventroposterior margin of coracoid: 0 - absent 1 - present

84. Cristospine:

0 - absent

1 - shallow and elongated 2 - deep and shortFORELIMB85. Proportional length of the humerus relative to the metacarpal IV (hu/mcIV): 0 - hu/mcIV > 2.50 1 - 1.50 < hu/mcIV < 2.50 2 - 0.40 < hu/mcIV < 1.50 3 - hu/mcIV < 0.4086. Proportional length of the humerus relative to the femur (hu/fe): 0 - hu/fe < 0.80 1 - 1.4 > hu/fe > 0.80 2 - hu/fe > 1.4087. Proportional length of the humerus plus ulna relative to the femur plus tibia (hu+ul/fe+ti): 0 - humerus plus ulna about 0.80% or less of femur plus tibia length (hu+ul/fe+ti < 0.80) 1 - humerus plus ulna larger than 0.80% of femur plus tibia length (hu+ul/fe+ti > 0.80)88. Pneumatic foramen on the ventral side of the proximal part of the humerus: 0 - absent 1 - present

89. Pneumatic foramen present on dorsal side of the proximal part of the humerus: 0 - absent 1 - present

90. Deltopectoral crest of the humerus: 0 - reduced, positioned close to the humerus shaft 1 - enlarged, proximally placed, with almost straight proximal margin 2 - enlarged, hatchet shaped, proximally placed 3 - enlarged, hatched shaped, positioned further down the humerus shaft 4 - enlarged, warped 5 - long, proximally placed, curving ventrally91. Medial (= ulnar) crest of the humerus: 0 - absent or reduced 1 - present, directed posteriorly 2 - present, massive, with a developed proximal ridge92. Distal end of the humerus: 0 - oval or D-shaped 1 - subtriangular93. Proportional length of the ulna relative to the metacarpal IV (ul/mcIV): 0 - ulna 3.6 times longer than metacarpal IV (ul/mcIV > 3.6) 1 - length of ulna between 3.6 and two times the length of metacarpal IV (3.6 > ul/mcIV > 2)

2 - ulna between two times and the same length of metacarpal IV

(2 > ul/mcIV > 1)

3 - ulna about the same length or smaller than metacarpal IV (ul/mcIV < 1)94. Diameter of radius and ulna: 0 - subequal 1 - diameter of the radius about half that of the ulna 2 - diameter of the radius less than half that of the ulna95. Distal syncarpals, shape: 0 - irregular 1 - from a rectangular unit 2 - form a triangular unit96. Pteroid: 0 - absent 1 - shorter than half the length of the ulna 2 - longer that half the length of the ulna97. Metacarpals I - III: 0 - articulating with carpus 1 - metacarpal I articulates with carpus, metacarpals II and III reduced 2 - not articulating with carpus98. Proportional length of the first phalanx of manual digit IV relative to the metacarpal IV (ph1d4/mcIV): 0 - both small and reduced 1 - both enlarged with ph1d4 over four times the length of mcIV (ph1d4/mcIV>4.0) 2 - both enlarged with ph1d4 about or less than two times the length of mcIV (ph1d4/mcIV<2.0) about 2 or smaller.

99. Proportional length of the first phalanx of manual digit IV relative to the tibiotarsus (ph1d4/ti): 0 - ph1d4 reduced 1 - ph1d4 elongated and less than twice the length of ti (ph1d4/ti smaller than 2.00) 2 - ph1d4 elongated about or longer than twice the length of ti (ph1d4/ti subequal/larger than 2.00)100. Proportional length of the second phalanx of manual digit IV relative to the first phalanx of manual digit IV (ph2d4/ph1d4): 0 - both short or absent 1 - elongated with second phalanx about the same size or longer than first (ph2d4/ph1d4 larger than 1.00) 2 - elongated with second phalanx up to 30% shorter than first (ph2d4/ph1d4 between 0.70 - 1.00) 3 - elongated with second phalanx more than 30% shorter than first (ph2d4/ph1d4 smaller than 0.70)101. Proportional length of the third phalanx of manual digit IV relative to the first phalanx of manual digit IV (ph3d4/ph1d4): 0 - both short or absent 1 - ph3d4 about the same length or larger than ph1d4 2 - ph3d4 shorter than ph1d4102. Proportional length of the third phalanx of manual digit IV relative to the second phalanx of manual digit IV (ph3d4/ph2d4): 0 - both short or absent 1 - ph3d4 about the same size or longer than ph2d4 2 - ph3d4 shorter than ph2d4103. Proportional length of the forth phalanx of manual digit IV relative to the first phalanx of manual digit IV (ph4d4/ph1d4): 0 - both short or absent 1 - both elongated, with the forth phalanx the longer than the first (ph4/d4/ph1d4>1.00)

2 - both elongated with the forth phalanx the same length or shorter, but longer than 35% the length of the first (1.00>ph4d4/ph1d4>0.35)

3 - both elongated with the forth phalanx less than 35% the length of the first (ph4d4/ph1d4<0.35)HINDLIMB104. Proportional length of the femur relative to the metacarpal IV (fe/mcIV): 0 - femur about twice or longer than metacarpal IV (fe/mcIV > 2.00) 1 - femur longer but less than twice the length of metacarpal IV (1.00 < fe/mcIV < 2.00) 2 - femur about the same length or shorter than metacarpal IV (fe/mcIV < 1.00)105. Length of metatarsal III: 0 - more than 30% of tibia length 1 - less than 30% of tibia length106. Fifth pedal digit: 0 - with four phalanges 1 - with 2 phalanges 2 - with 1 or no phalanx (extremely reduced)107. Last phalanx of pedal digit V: 0 - reduced or absent 1 - elongated, straight 2 - elongated, curved 3 - elongated, very curved (boomerang shape)

DATA MATRIX

***Ornithosuchus longidens***0000000-00 000000---0 0000----00 0000--0 -00 0000000000 10000--000 0000000000 0000000000 0000000000 0000000000 0000000***Herrerasaurus ischigualastensis***0000000-00 000000---0 0000----00 0000--0-00 0000000000 00000--000 0000000000 0000000000 0000000000 0000000000 0000020

***Scleromochlus taylori*** 000?0?0-?0 ?000?0---0 0000----?? ???0--??00 0?????0??? ??0?0--00? 0?0??0??00 ??000????0 0????00??0 ???0?0???? ??0????

***Anurognathus ammoni*** 010001???0 ?????0---0 00?0----0? ?0?0--0-?? ??????0??? 10000--000 0110?0??0? ??100??0?? ????011??1 0000?1011? ???001?

***Rhamphorhynchus muensteri***0010100-00 000010---0 0000----00 0000--0-01 1000000110 11010--000 0100000000 0001000001 0001111002 0011?10122 2221012

***Darwinopterus modularis***

0010101000 0000112000 0011010?00 1000--0-01 1?0?0?0??? ?0000--000 0120?00010 000?0????? ????1????? ??1????21? ???????

***Pterodactylus antiquus***0010101000 000010---0 0011001000 1000--0-12 1?0?000??? ?1000--000 0120000011 1010000001 0001211005 0021?10212 2222020

***Nyctosaurus gracilis***0010101000 0000?0---0 0010----00 1000--0-0? 110?000121 02000--300 0100111001 0010000001 0001311103 0?31222222 22?20??

***Nemicolopterus crypticus*** 0010101000 0000?0---0 0011110001 1000--0-0? 2?????0??? ?1000--300 0100?0?001 ??100000?? ?0???11?15 ??2????21? 22?2120

***Pteranodon longiceps***1020101010 300010---0 001112-000 1101221101 2101000121 02000--300 0100111101 0110111012 0001211104 1131222222 2232020

***Istiodactylus latidens***0010101100 2???10---0 0?11110?01 21?0--0-01 2?01?00??1 ?0000--200 0101?1110? 01???11102 0002?1?014 11?22????? ???????

***Nurhachius ignaciobritoi***0010101100 ?000?0---0 001?????01 110??????1 2?????0??? ?000--2000 10101??010 1???1?1020 0?22110?41 ?222?1212? ??21??

***Tropeognathus mesembrinus***0010101000 3000110200 101?????00 1101001001 2101020121 0100101000 0100?????? ?????????? ?????????? ?????????? ???????

***Anhanguera santanae***0010101010 3000110200 1011100100 1101001001 2101010121 0100??1001 0100101101 011??21102 100????014 11?22?1??? ???????

***Anhanguera blittersdorffi***0010101010 3000110200 101?????00 1101001001 2101010121 0100101001 0100?????? ?????????? ?????????? ?????????? ???????

***Anhanguera piscator***0010101010 3000110200 1011100100 1101001001 21010?0121 0100101001 0100101101 0110121112 1002211014 11222?1??? ???2120

***Ludodactylus sibbicki***0010101000 000010---0 ?011100100 01011?1101 110?0?0??? ?1000--001 0100?????? ?????????? ?????????? ?????????? ???????

***Dsungaripterus weii***2010101000 1101111100 0110----00 1001211101 2111110121 01000--110 1100111101 0010?000?? ?0?2210??5 ?0311?1212 22?2120

***"Phobetor" parvus***

0010101000 1101111100 0110----00 1001211101 2?1?1?0??? ?1000--110 1100?????? 0????????? ?????????? ????1????? ???????

***Quetzalcoatlus sp.***0010101000 3010?14?00 0010----00 100??????1 21???001?? 02000--300 0100111021 22?0?00002 010?20?105 20311??213 22?2?20

***Azhdarcho lancicollis***?????????? 3????????? ?????????? ?0???????? ?????????? ???????300 010?111021 22???????? ???????105 ?0???????? ???????

***Zhejiangopterus linhaiensis***0010101000 3010?0---0 0010----00 1000--0-01 2?1???0??? ?2000--300 010011??21 22?0?000?? ?10?200??5 ??31?2?213 ???2???

***Chaoyangopterus zhangi*** 1010101??? ?0???????0 00???????? ?????????? ??????0??? ?2000--300 0100?01011 00???000?? ?00?200??? ??31?2?212 22321?0

***Shenzhoupterus chaoyangensis*** 1010101101 301020---0 001?????0? 2001241101 2?1???0??? ?2000--300 0100?01011 ?????0??0? ?0??200??? ??31???212 22321??

***Tupuxuara leonardii***0010101100 3010213400 001?????01 1001231101 2111130121 0200112300 0100111101 00???00002 0012210105 20311?1213 ???2???

***Thalassodromeus sethi***0010101100 3010213400 001112-001 1001231101 2111130121 ??000--300 0100?????? ?????????? ?????????? ?????????? ???????

***Tupandactylus imperator***

0011101100 3010213301 001??????1 1001221101 2?1??????? ?1??113300 0100?????? ?????????? ?????????? ?????????? ???????

***Europejara olcadesorum***?01??????? ????2????? ?01??????? ????????01 20?????1?1 ?110113300 0100?????? ?????????? ?????????? ?????????? ???????

***Caiuajara dobruskii*** **nov gen., nov sp.**

00111011?? 3010213301 00???????1 0????????? 20???01??? 1110113300 0000?0110? 00?0?00002 ?01?21?115 203112???? ???????

***Tapejara wellnhoferi***0011101100 3010213300 0011111011 1001221101 2011101121 1110113300 0100?01101 00?0?00002 001?210115 203112?21? ???2120

***Sinopterus dongi*** 0011101100 3010?13300 0011100011 1011211101 2?1???1??? ??10114300 0100?01011 00???00002 00??2111?5 2?31?2?212 22321??

***Eopteranodon* *lii***

001110110? ?????13300 00???????1 1????????? ?1???0???? ?1?0114300 0000??1??? ?0?????0?? ????211??5 ??31?2?212 22?2???

**"*Huaxiapterus*" *corollatus***0011101??? ?????1?310 001??????? ?????????? ?????????? ?10114300 0100?01011 ?????000?? ?0??211??5 ??3????213 22321??

**"*Huaxiapterus*" *benxiensis***0011101100 ?0???13310 001??????1 10?1211101 2?1???1??? 1110114300 0100?01011 ?????????? ????2????? ??3??2?212 22?2120

***Aymberedactylus cearensis* gen. et sp. nov.**

?0???????? ?????????? ?????????? ?????????? ?????????? 1100114300 0000?????? ?????????? ?????????? ?????????? ???????

**Table A) Measurements of the mandible of *Aymberedactylus cearensis* gen. et sp. nov.**

| Structure | Measurement (mm) |
| --- | --- |
| Mandible length (from rostral tip to the point between the two glenoid fossae) | 252 |
| Mandible length (from rostral tip to the tip of retroarticular process) | 269 |
| Mandibular symphysis length | 143 |
| Mandibular symphysis depth (preserved) | 13 |
| Mandibular rami length | 134 |
| Mandibular rami depth | 15 |
| Retroarticular process length | 18 |
| Inter-glenoid fossae distance | 80 |

**Figure A) Measurement of mandibular width (new character 51)**. Red line indicates the measurement of mandibular width, in between the articulation surfaces for the quadrates including their own widths.


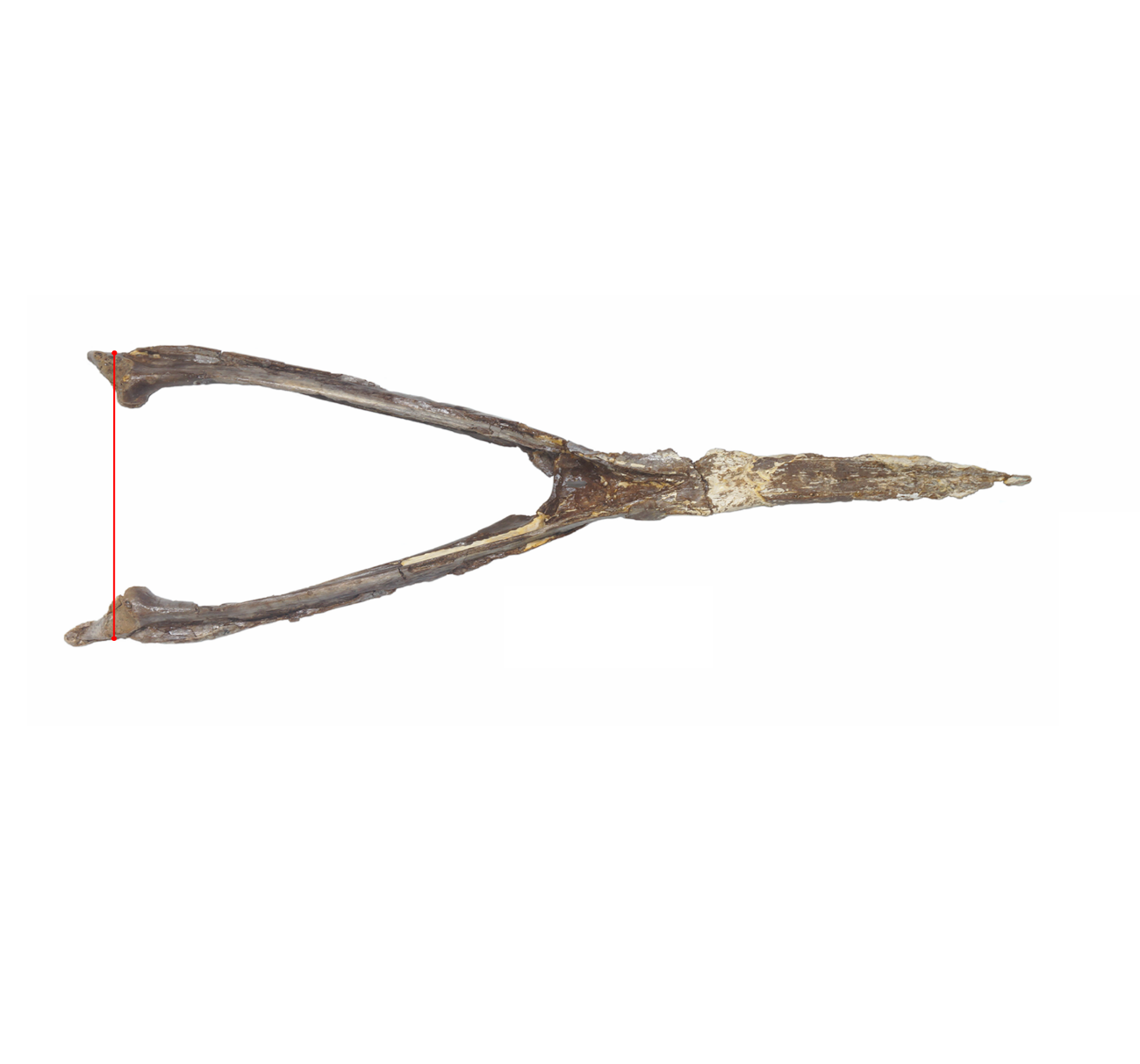

Supplement: S1 File — List A. Phylogenetic Analysis. Character list and data matrix. Table A. Measurements of Aymberedactylus cearensis gen. et sp. nov. Figure A. Measurement of mandibular width (new character 51). (DOCX) [file pone.0162692.s001.docx]
